# Loss of CD14 leads to disturbed epithelial-B cell crosstalk and impairment of the intestinal barrier after *E. coli* Nissle monoassociation

**DOI:** 10.1038/s41598-017-19062-7

**Published:** 2018-01-15

**Authors:** Marijana Basic, Manuela Buettner, Lydia M. Keubler, Anna Smoczek, Inga Bruesch, Stephanie Buchheister, André Bleich

**Affiliations:** Institute for Laboratory Animal Science, Hannover Medical School, 30625 Hannover, Germany

## Abstract

The TLR4 co-receptor CD14 was identified as an IBD candidate gene. Here, its influence on the intestinal barrier was addressed utilizing *E. coli* Nissle (EcN), which induces severe inflammation in germfree TLR4^−/−^ mice. After monoassociation, EcN was detected in spleens and livers of TLR4^−/−^ and CD14^−/−^ but not wildtype mice. Barrier impairment was characterized by increased apoptosis and decreased epithelial junction (EJ) expression and was reversed by TLR2 stimulation in CD14^−/−^ mice. Bone marrow (BM) transplantation revealed contribution of hematopoietic and non-hematopoietic cells towards intestinal homeostasis. EcN inoculated WT mice showed B cell activation, CD14^−/−^ and TLR4^−/−^ mice cytotoxic T cell and impaired B cell responses. The latter was characterized by absence of B cells in TLR4^−/−^ mice, decreased levels of EcN induced immunoglobulins and downregulation of their transporter pIgR. EcN colonization of mice with genetically or antibody induced impaired B cell response resulted in dissemination of EcN and downregulation of EJ. BM chimeras indicated that CD14 originating from radiation resistant cells is sufficient to restore EJ-function. Overall, CD14/TLR4 signalling seems to be critical for intestinal barrier function and for the crosstalk between B cells and the epithelium, underlining that CD14 serves as a protective modulator of intestinal homeostasis.

## Introduction

The gastrointestinal tract is colonized by a complex community of microorganisms, some of which are beneficial or potentially pathogenic^1,2^. The intestinal barrier is composed from physical, cellular and chemical components^3^. This efficient barrier separates the luminal content from the host tissues, mediates interaction between intestinal immune cells and the gut microflora and regulates absorption of nutrients^4–6^. Intestinal epithelial cells (IEC) play a central role in the intestinal barrier maintenance^6^. These cells build a monolayer kept tightly together by epithelial junctions (EJ) such as tight (TJ) or adherens (AJ) junctions, which among other functions prevent translocation of luminal bacteria^7,8^. IEC and lamina propria (LP) immune cells recognize luminal antigens mainly by pattern recognition receptors (PRRs) such as toll like receptors (TLRs). TLRs, as part of the innate immune system, have a key role in maintaining the integrity of the intestinal barrier and promoting the maturation of the mucosal immune system^9,10^. Antigen recognition activates the PRR downstream cascades, which results in the expression of anti-inflammatory or pro-inflammatory cytokines and antimicrobial or antiviral mediators^11^. The intestinal homeostasis is shaped by multifaceted interactions between the gut microflora, the intestinal epithelium and the host immune system. This delicate system can be disrupted by bacterial imbalance, defects in the epithelial barrier or/and immune regulation mechanisms and subsequently lead to the development of inflammatory bowel disease (IBD)^12–14^. IBD, with the two main forms Crohn’s disease (CD) and ulcerative colitis (UC), is a chronic multifactorial gastrointestinal inflammatory disorder. It is mostly a disease of the developed world, although its incidence is increasing worldwide^15^. The exact mechanisms that underlie IBD development are not fully understood yet. Nevertheless, IBD results due to genetic predisposition (susceptibility) and an exaggerated immune response to the enteric microflora^16^.

CD14 is a PPR for a variety of bacterial cell wall products such as lipopolysaccharide (LPS) and lipoprotein, and an important co-receptor of the TLR4 and TLR2 signalling pathway. It is expressed by myeloid lineage cells such as monocytes and macrophages or on non-myeloid lineage cells such as IEC as a receptor anchored in the cell membrane (mCD14) or secreted as soluble CD14 (sCD14)^17–20^. The predominant form of CD14 in the gut is sCD14 that is released by IEC, whereas expression of mCD14 on macrophages and IECs in the healthy gut is very low^18^. In animal models of experimental colitis *Cd14* has been identified as a promising candidate gene^21^, which plays a protective role in experimental IBD^18,22^. In addition, human and mouse *Cd14* promoter polymorphisms are discussed to be associated with IBD^23–25^. Moreover, sCD14 seems to contribute to the host defence against bacterial infections^26–28^.

*E. coli* Nissle 1917 (EcN) is a Gram-negative probiotic bacterium, first isolated by Dr. A. Nissle^29^. This bacterium was shown to ameliorate experimental colitis^30,31^ and to maintain remission of UC in patients^32^. However, it was shown that it also induces severe and lethal inflammation in germfree (GF) C3H/HeJZtm mice carrying a defective *Tlr4* gene spreading beyond the gut^33^. Therefore, in the present study EcN monoassociation was utilized in a CD14^−/−^ mouse model to reveal alterations of the intestinal mucosa and the influence of CD14 on the intestinal homeostasis.

## Results

### GF mice lacking TLR4 and CD14 display bacterial translocation and intestinal barrier impairment after EcN monoassociation

**Figure 1:**
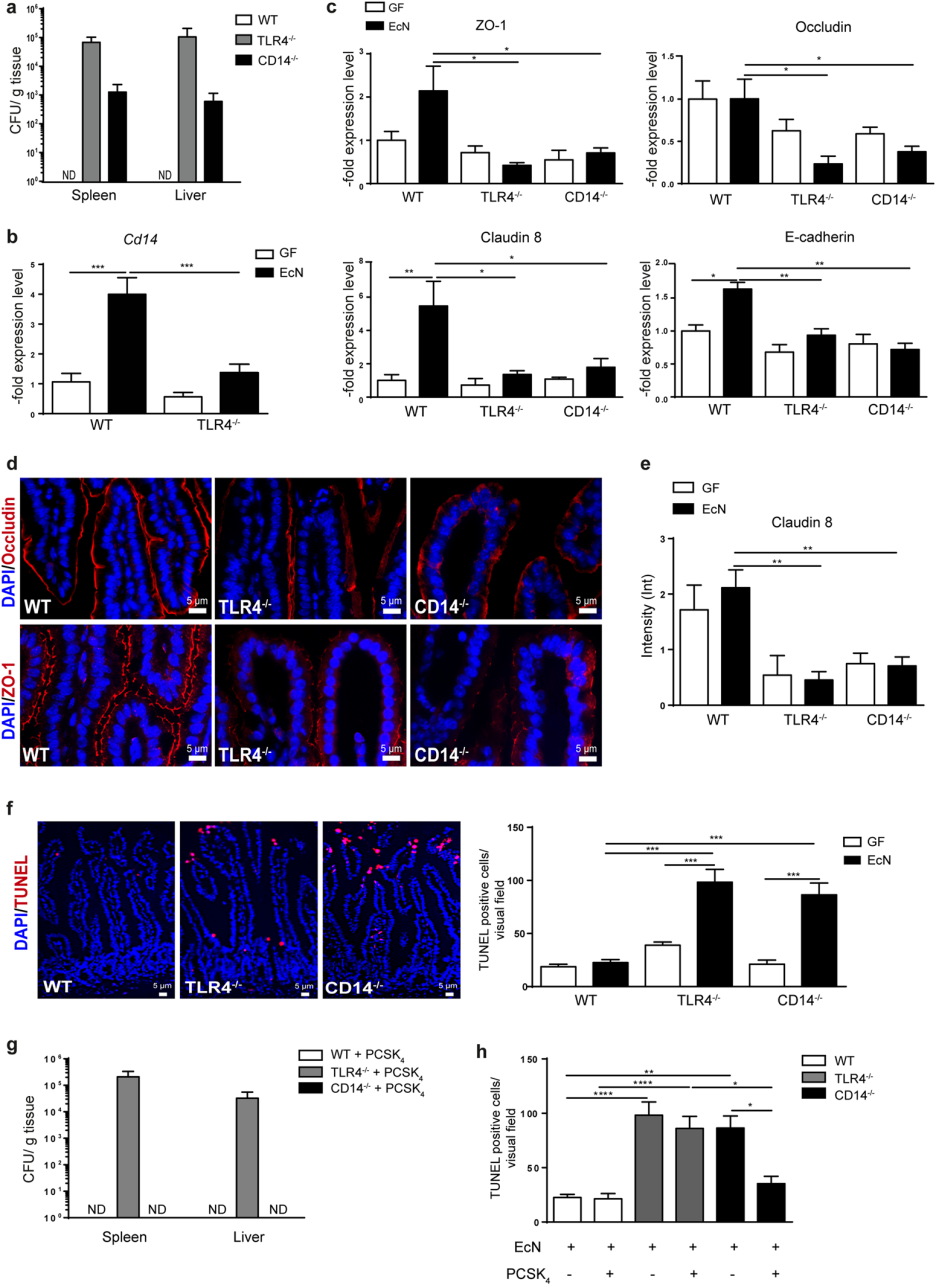
Intestinal epithelial barrier impairment in CD14^−/−^ and TLR4^−/−^ mice after EcN monoassociation. (**a**) Bacterial CFU were determined in WT, TLR4^−/−^ and CD14^−/−^ mice 72 h p.i. in spleen and liver (n = 4–5). ND-not detected. (**b**) Gene expression of CD14 measured by qPCR in total RNA isolated from ileum of WT and TLR4^−/−^ mice. Relative differences in gene expression were calculated by the comparative 2^−ΔΔCt^ method. Values are shown as fold induction (n = 4–5). (**c**) Gene expression of TJ (ZO-1, occludin and claudin 8) and AJ (E-cadherin) measured by qPCR in total RNA isolated from ileum. Relative differences in gene expression were calculated by the comparative 2^−ΔΔCt^ method. Values are shown as fold induction (n = 4–5). (**d**) Representative images of immunofluorescence staining of occludin (red) and ZO-1 (red) in the distal ileum obtained from WT, TLR4^−/−^ and CD14^−/−^ mice 72 h post EcN monoassociation. Nuclei (blue) were counterstained with DAPI (n = 4). (**e**) Densitometric quantification of claudin 8 protein expression measured in ileal lysates before and after EcN colonization normalized to GAPDH. Blot images were analysed by Image Lab^TM^ Software (n = 4–5). (**f**) Representative images of TUNEL positive cells (red) in the IEC barrier of WT, TLR4^−/−^ and CD14^−/−^ mice 72 h after EcN monoassociation. Nuclei (blue) were counterstained with DAPI. Quantification of TUNEL positive cells in the small intestine before and after EcN colonization (n = 4–5). (**g**) Bacterial CFU were determined 72 h after PCSK_4_ and EcN stimulation in spleen and liver of WT, TLR4^−/−^ and CD14^−/−^ mice (n = 5–6). ND-not detected (**h**). Quantification of TUNEL positive cells in the small intestine after PCSK_4_ stimulation and EcN colonization (n = 4–6).

In contrast to wildtype (WT) mice, EcN monoassociation resulted in increased bacterial invasion in TLR4^−/−^ mice to liver and spleen 3 days after inoculation (Fig. 1a). CD14^−/−^ mice also displayed bacterial translocation to these organs but to a lower extent. However, EcN capacity to colonize the gut was similar among all three strains (Supplementary Fig. S1a). In WT mice EcN monoassociation resulted in strongly increased CD14 gene expression, but not in TLR4^−/−^ mice (Fig. 1b). As bacterial dissemination beyond the intestine may be the result of an impaired epithelial barrier, the expression of epithelial junctions (EJ) and the epithelial cell death rate in the gut was determined. EcN monoassociation led to an upregulated gene expression of zonula occludens 1 (ZO-1) and claudin 8 (TJ representative), and E-cadherin (AJ representative) in WT mice (Fig. 1c). However, gene expression of the TJ protein occludin was not upregulated after EcN stimulation. In both, TLR4^−/−^ and CD14^−/−^ mice, EcN monoassociation did not upregulate gene expression of any of these EJ. Similarly, immunostaining of the distal ileum showed continuous expression of occludin and ZO-1 along the crypt-villus axis in WT mice (Fig. 1d), whereas in TLR4^−/−^ and CD14^−/−^ mice the staining signal was poor or diffuse after EcN monoassociation. The protein expression of claudin 8 was significantly lower in TLR4^−/−^ and CD14^−/−^ mice than in WT after EcN challenge, as shown by densitometric analysis of western blot data (Fig. 1e).

In addition, the number of TUNEL positive cells in the epithelial layer was increased in TLR4^−/−^ and CD14^−/−^ mice, but not in WT mice after EcN challenge (Fig. 1f). Thus, increased cell death and reduced EJ expression point towards epithelial barrier impairment in this model.

### TLR2 stimulation prevents bacterial dissemination in mice lacking CD14, but not in mice lacking functional TLR4 receptor

To analyse whether TLR2 stimulation prevents bacterial translocation by restoring epithelial barrier tightness, mice were challenged with TLR2 agonist PCSK_4_. Stimulation of TLR2 showed no effect in TLR4^−/−^ mice as bacteria were found in all organs. However, in CD14^−/−^ mice EcN was not detected any more in spleen and liver (Fig. 1g). PCSK_4_ stimulation did not affect the ability of EcN to colonize the gut (Supplementary Fig. S1b).

TLR2 stimulation strongly increased gene expression of E-cadherin, ZO-1, occludin and claudin 8 in WT and CD14^−/−^ mice, but not in TLR4^−/−^ mice. Unexpectedly, during simultaneous TLR2 stimulation and EcN challenge the expression of epithelial junctions decreased in all three mouse strains (Supplementary Fig. S2). Interestingly, the number of dead cells was significantly decreased in CD14^−/−^ mice after TLR2 and EcN stimulation and comparable to the number of dead cells in WT mice. In contrast, the cell death rate stayed high in TLR4^−/−^ mice (Fig. 1h). Even though expression of EJ in TLR4^−/−^ and CD14^−/−^ mice was comparable after TLR2 and EcN stimulation, the cell death rate was significantly lower in CD14^−/−^ mice indicating that the CD14-independent TLR2 stimulation strengthens the intestinal barrier and prevents bacterial translocation, but that the protective TLR2 stimulation cannot compensate for the TLR4 deficiency.

### Host immune response towards EcN colonization

To differentiate the contribution of epithelial cells and LP hematopoietic cells towards barrier maintenance, GF bone marrow (BM) chimeras were studied. Thus, recipient mice of all three strains were irradiated, supplemented with BM of WT, TLR4^−/−^ or CD14^−/−^ mice and inoculated with EcN 10 weeks later. The migration of EcN into spleen and liver of TLR4^−/−^ BM chimeras was very high, whereas no or low dissemination was seen in WT BM chimera. Irradiated WT mice, which received hematopoietic cells from TLR4^−/−^ or CD14^−/−^ mice, displayed higher bacterial translocation to spleen and liver than WT BM controls (Supplementary Fig. S3). In addition, no bacterial spread to the liver was observed in irradiated WT mice that received hematopoietic cells from CD14^−/−^ mice. In TLR4^−/−^ and CD14^−/−^ irradiated mice receiving WT BM cells, bacterial dissemination to spleen and liver was higher than in WT BM controls, but lower than in susceptible TLR4^−/−^ BM controls. Together these findings indicate the importance of the hematopoietic and non-hematopoietic cell compartment to reduce bacterial invasion and maintain the intestinal barrier in this model.

**Figure 2:**
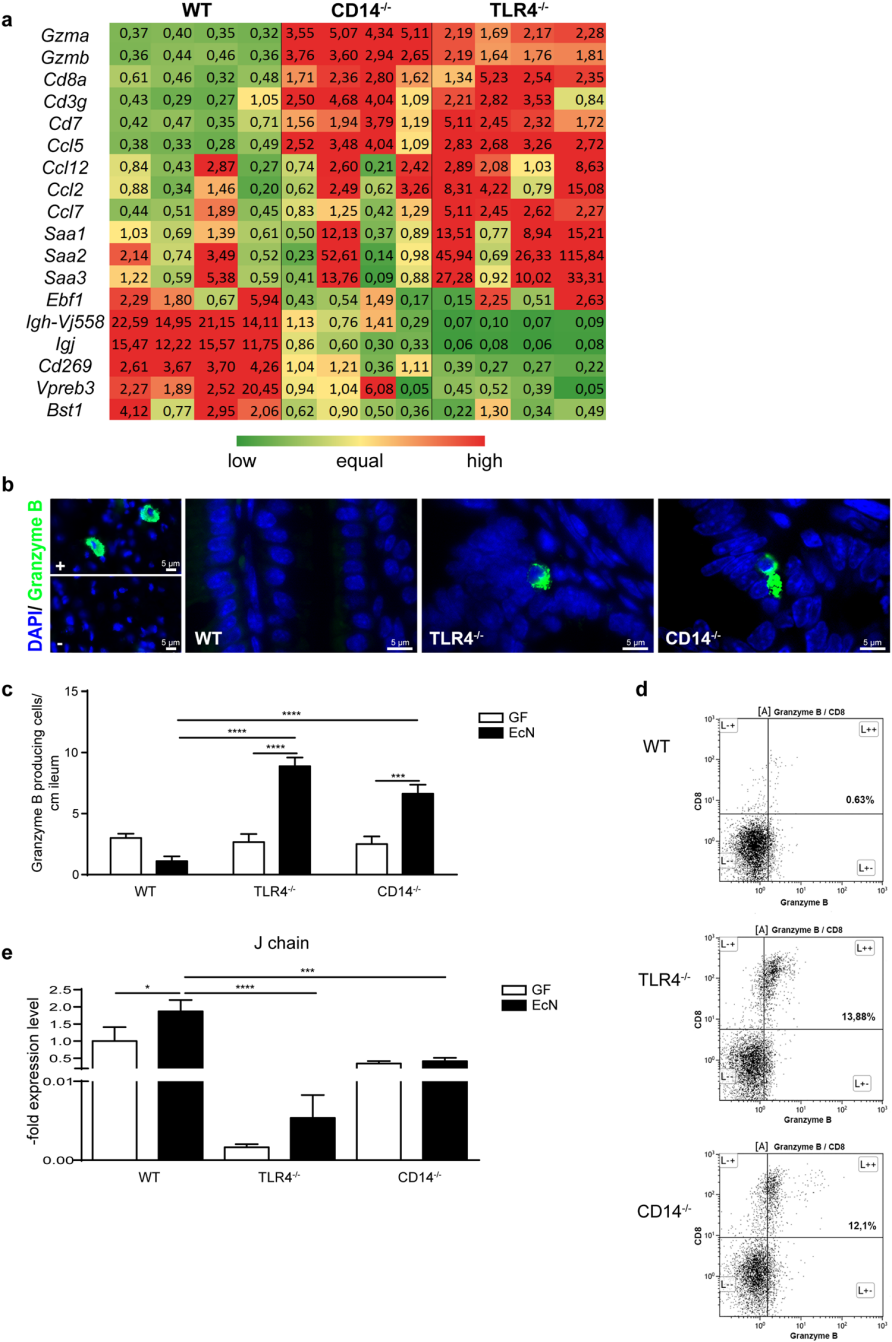
Host immune response to EcN colonization. (**a**) Gene expression was determined by microarray analysis in the small intestine of WT, TLR4^−/−^ and CD14^−/−^ mice 72 h after EcN monoassociation. The heat map presents “up” (red) and “down” (green) regulated genes (n = 4) in comparison to WT gene expression values after EcN challenge. Numbers within the table represent fold change calculated based on the gene expression. (**b**) Representative images of immunofluorescence staining for granzyme B positive cells (green) in the small intestine 72 h after EcN colonization from WT, TLR4^−/−^ and CD14^−/−^ mice, including positive (+; mouse nasopharyngeal-associated lymphoreticular tissues - NALT) and negative control (−). Nuclei (blue) were counterstained with DAPI (n = 4). (**c**) Quantification of granzyme B positive cells from the distal ileum before and after EcN challenge (n = 4–5). (**d**) Representative flow cytometry dot plot analysis of granzyme B + CD3 + CD8 + cells isolated from the small intestinal lamina propria of WT, TLR4^−/−^ and CD14^−/−^ mice (n = 3). (**e**) Gene expression of J chain, measured by qPCR in the small intestine of WT, TLR4^−/−^ and CD14^−/−^ mice before and after EcN stimulation. Relative differences in gene expression were calculated by the comparative 2^−ΔΔCt^ method. Values are shown as fold induction (n = 4–5).

Considering the BM chimeras’ data, the contribution of the immune cell compartment for the barrier maintenance in the gut was investigated by performing microarray analysis utilizing ileal samples (Fig. 2a). In WT mice genes involved in B cell activation and differentiation were upregulated, such as joining chain (J chain), which has been shown to be important for the transport of immunoglobulin A (IgA) and IgM through mucosal membranes via polymeric immunoglobulin receptor (pIgR)^34^. The J chain gene was found to be more than 10 times higher expressed in WT mice compared to CD14^−/−^ and TLR4^−/−^ mice after EcN challenge. In contrast, in CD14^−/−^ and TLR4^−/−^ mice upregulated genes were involved in the cytotoxic T cell mediated immune response such as granzyme A and B.

**Figure 3:**
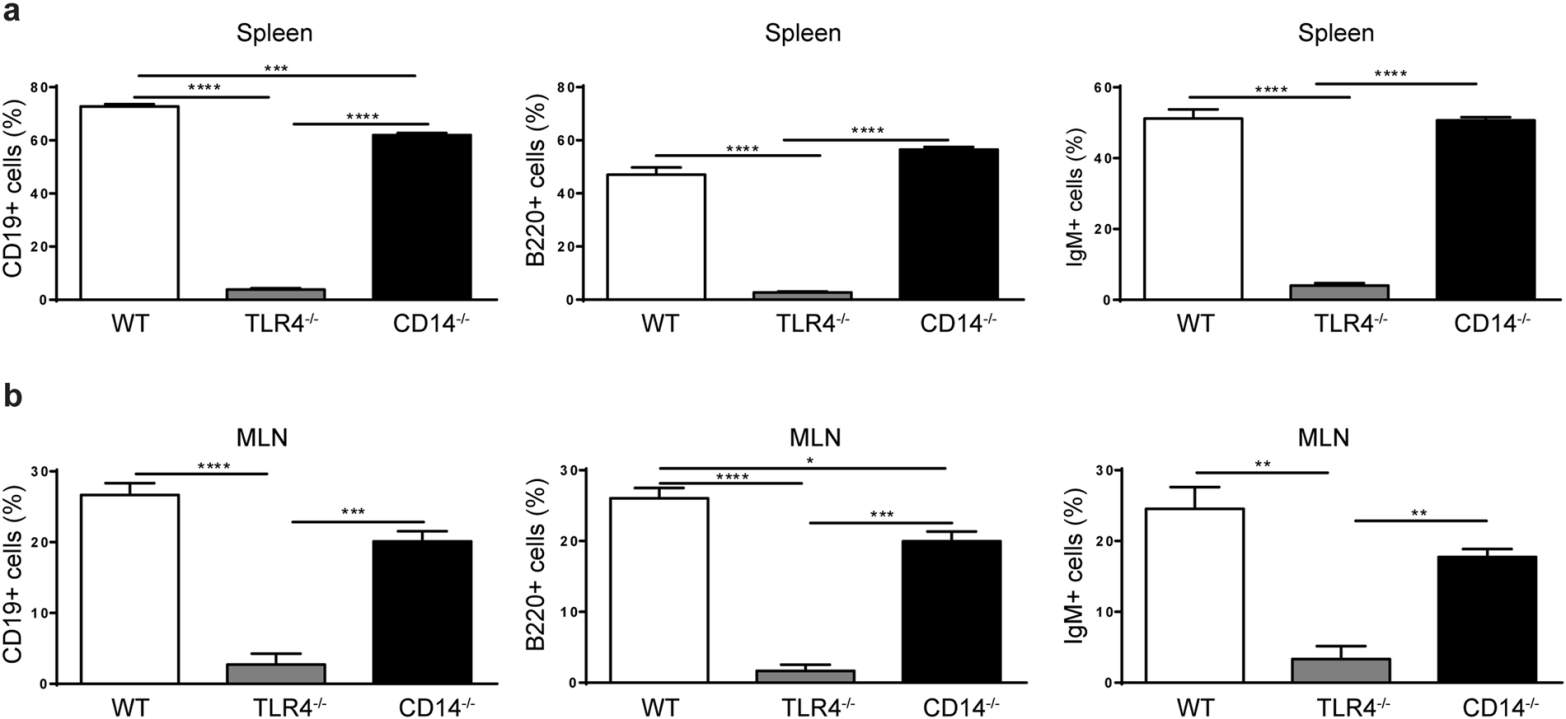
Absence of B cells in TLR4^−/−^ mice. (**a**) Flow cytometry analysis of CD19+, B220+ and IgM+ positive cells isolated from spleens of germfree WT, TLR4^−/−^ and CD14^−/−^ mice (n = 3). (**b**) Flow cytometry analysis of CD19+, B220+ and IgM+ positive cells isolated from MLN of germfree WT, TLR4^−/−^ and CD14^−/−^ mice (n = 3).

These observations were verified by real time PCR, immunohistological staining or flow cytometry. More granzyme producing cells were seen in CD14^−/−^ and TLR4^−/−^ mice than in WT mice after immunostaining (Fig. 2b,c). Granzyme B positive cells found in the small intestine were mostly CD3+ CD8+ cells as confirmed by flow cytometry (Fig. 2d). In contrast, J chain was found to be upregulated after EcN stimulation only in WT mice (Fig. 2e). Furthermore, staining splenic and MLN cells with anti-CD19, anti-B220 and anti-IgM antibodies revealed presence of B cells in WT and CD14^−/−^ mice, but virtually none in TLR4^−/−^ mice (Fig. 3a,b).

Thus, these results indicate that B cells might play an important role in the regulation of the intestinal barrier and that the CD14/TLR4 pathway is critical for this defence mechanism against invading pathobionts.

### Antibody production and transport trough pIgR relies on an intact CD14/TLR4 signalling pathway

**Figure 4:**
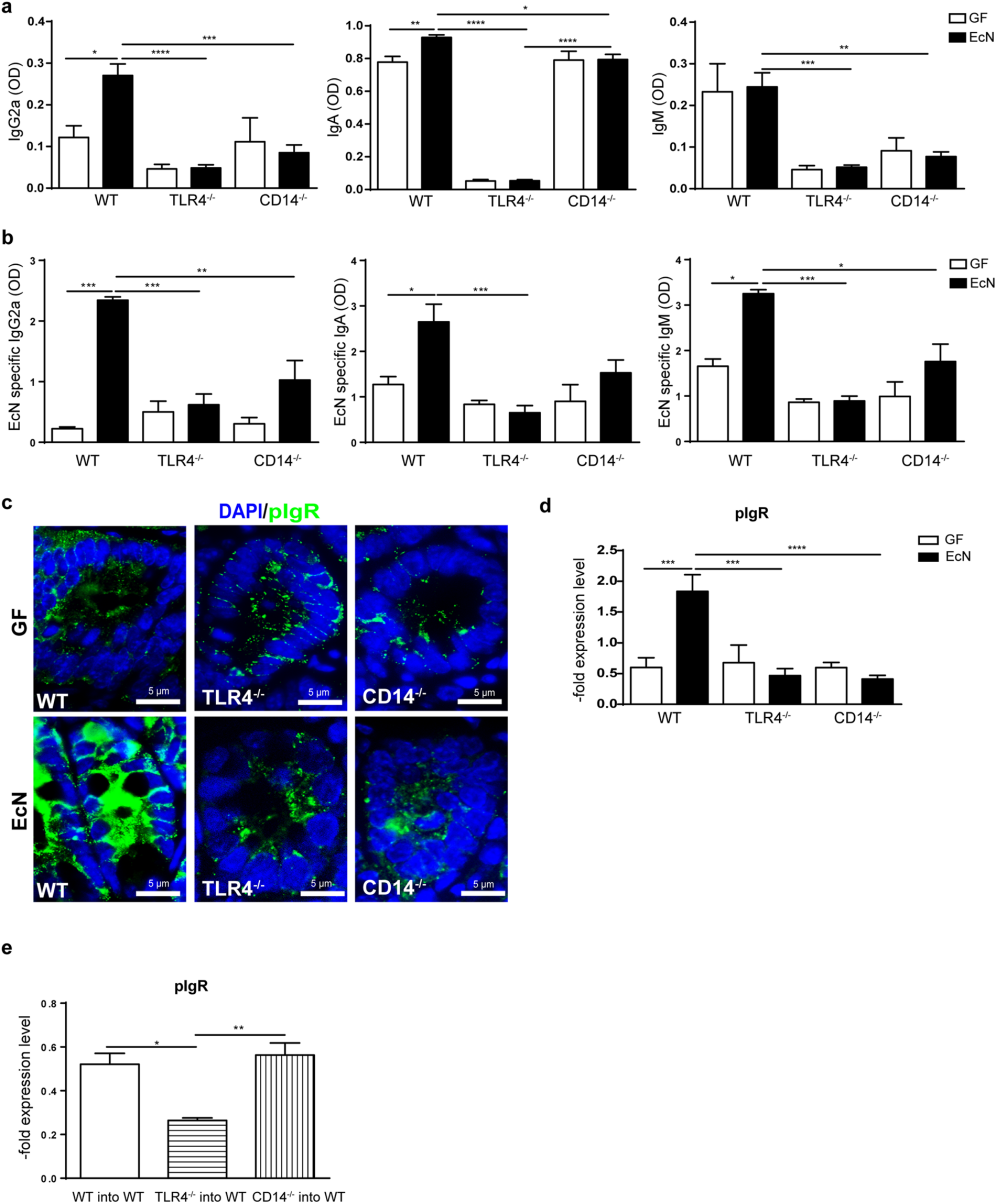
Antibody production and transport to the intestinal lumen. (**a**) Measurement of total IgG2a, IgA and IgM levels in the small intestinal lavage of WT, TLR4^−/−^ and CD14^−/−^ mice before and after EcN challenge (n = 5–6). (**b**) Measurement of EcN specific IgG2a, IgA and IgM levels in the small intestinal lavage of WT, TLR4^−/−^ and CD14^−/−^ mice before and after EcN challenge (n = 5–6). (**c**) Representative images of immunofluorescence staining for pIgR (green) in the distal small intestine before and 72 h after EcN colonization from WT, TLR4^−/−^ and CD14^−/−^ mice. Nuclei (blue) were counterstained with DAPI (n = 4). (**d**) pIgR gene expression measured by qPCR in the small intestine of WT, TLR4^−/−^ and CD14^−/−^ mice before and after EcN infection. Relative differences in gene expression were calculated by the comparative 2^−ΔΔCt^ method. Values are shown as fold induction (n = 4–5). (**e**) pIgR gene expression measured by qPCR in the small intestine of bone marrow chimeras (irradiated WT mice, which received BM from WT, TLR4^−/−^ and CD14^−/−^ mice). Relative differences in gene expression were calculated by the comparative 2^−ΔΔCt^ method. Values are shown as fold induction (n = 5).

To further characterize B cell contribution, the levels of secreted IgA and IgM in the gut 72 hours post EcN inoculation were determined utilizing ELISA. WT mice produced higher levels of IgG2a, IgA and IgM (Fig. 4a) after EcN monocolonization than mice lacking CD14 or having a defective TLR4 receptor. Moreover, EcN increased expression of IgG2a and IgA in WT mice, but not in TLR4^−/−^ and CD14^−/−^ mice. In addition, specificity of antibodies towards EcN was determined. Interestingly, EcN stimulated the secretion of EcN specific antibodies in WT mice; however, no significant upregulation was observed in CD14^−/−^ and TLR4^−/−^ mice (Fig. 4b).

Antibodies produced within the LP are transported to the intestinal lumen across epithelial cells by the pIgR, expressed only on the basolateral surface of IEC^35^. Thus, the expression of the pIgR was analysed. GF mice showed very low expression of the pIgR, which was upregulated in WT mice after EcN monoassociation. This is opposite to TLR4^−/−^ and CD14^−/−^ mice, in which expression levels stayed unchanged (Fig. 4c,d). Furthermore, the expression of pIgR in BM chimeras after EcN challenge was investigated. In WT mice, which received BM cells from CD14^−/−^ mice, expression of pIgR was comparable to the WT control BM chimeras. In contrary, WT mice receiving TLR4^−/−^ BM, showed significantly lower pIgR expression than WT or CD14^−/−^ BM recipients (Fig. 4e).

These results indicate the importance of the intact TLR4 signalling pathway and its co-receptor CD14 for the expression of pIgR and the immunoglobulin transport to the intestinal lumen.

### B cells modulate epithelial function

**Figure 5:**
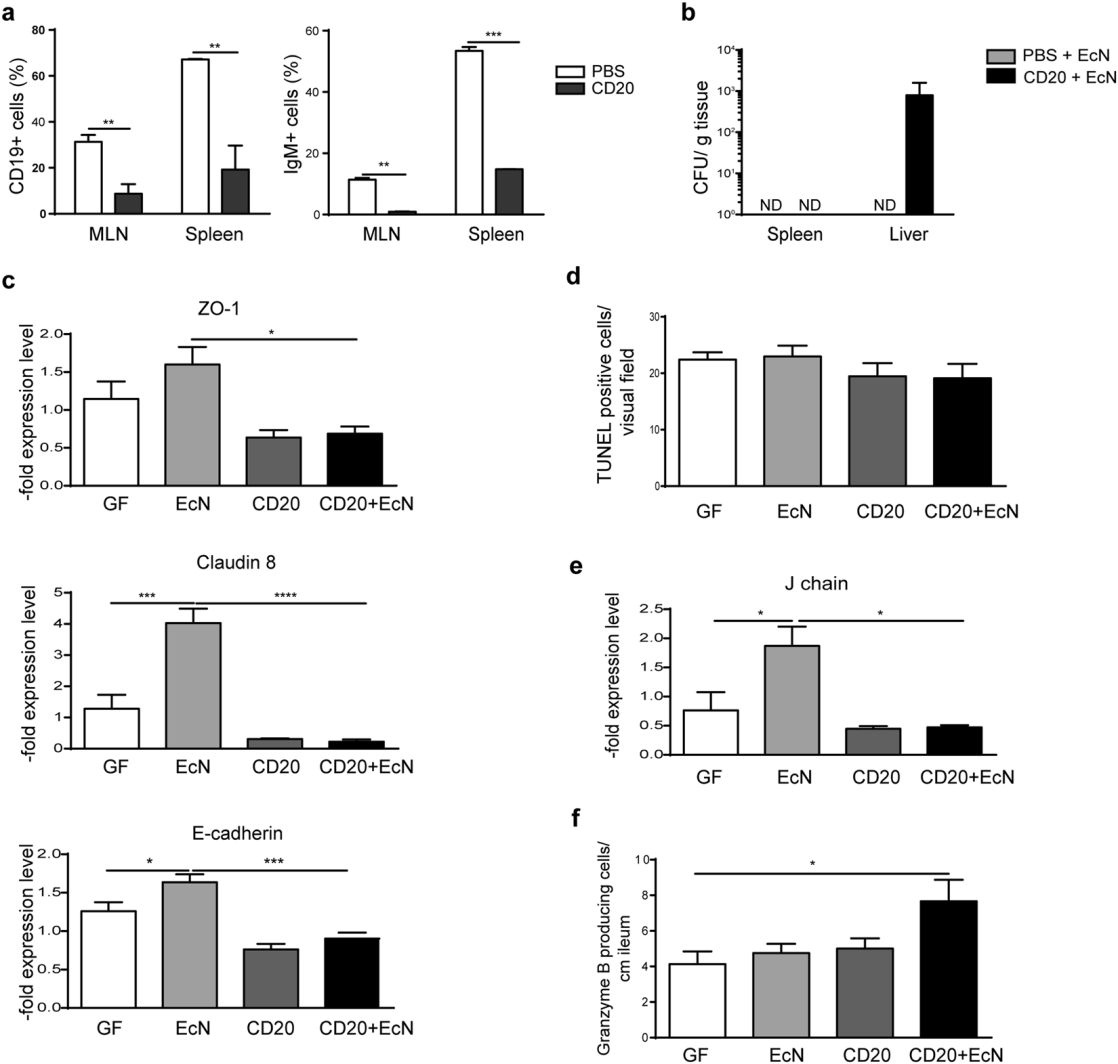
B cell depletion and impact on the intestinal barrier in WT mice. (**a**) Flow cytometry analysis of IgM+ and CD19+ positive cells isolated from MLN and spleen before and after CD20 treatment (n = 3, 5). (**b**) Bacterial CFU determined 72 h post EcN monoassociation with or without CD20 treatment in spleen and liver of WT mice (n = 5). ND-not detected (**c**). Gene expression of ZO-1, claudin 8 and E-cadherin measured by qPCR in total RNA isolated from WT ileum. Relative differences in gene expression were calculated by the comparative 2^−ΔΔCt^ method. Values are shown as fold induction (n = 5). (**d**) Quantification of TUNEL positive cells in the small intestine after CD20 treatment and EcN colonization (n = 5). (**e**) J chain gene expression measured by qPCR in the distal small intestine of WT mice. Relative differences in gene expression were calculated by the comparative 2^−ΔΔCt^ method. Values are shown as fold induction (n = 5). (**f**) Quantification of stained granzyme B positive cells in the WT ileum (n = 5).

To elaborate the B cell-barrier/epithelial cell interaction, B cells were depleted in WT mice using a CD20 antibody before EcN challenge. B cells were found to be reduced after CD20 administration (Fig. 5a). After B cell depletion, EcN disseminated beyond the intestine and was detected in the liver (Fig. 5b). However, B cell depletion did not influence EcN gut colonization rate (Supplementary Fig. 1c). Strikingly, B cell depletion abrogated EcN mediated upregulation of ZO-1, claudin 8 and E-cadherin gene expression (Fig. 5c). The rate of TUNEL positive cells in the gut remained unchanged (Fig. 5d). Furthermore, the expression of J chain was diminished, whereas the number of granzyme B producing cells was slightly increased (Fig. 5e,f).

**Figure 6:**
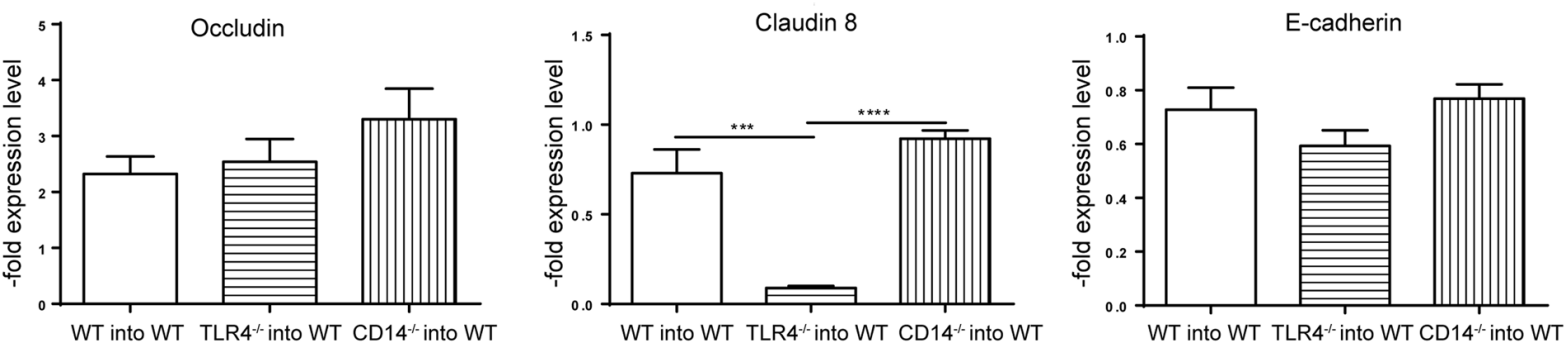
Impact of WT, TLR4^−/−^ and CD14^−/−^ bone marrow transplantation on the expression of epithelial junctions. Occludin, claudin 8 and E-cadherin gene expression measured by qPCR in total RNA isolated from ileum of WT, TLR4^−/−^ and CD14^−/−^ bone marrow transplanted WT mice after the challenge with EcN. Relative differences in gene expression were calculated by the comparative 2^−ΔΔCt^ method. Values are shown as fold induction (n = 5).

Moreover, as contrary to WT and CD14^−/−^ mice B cells were absent in TLR4^−/−^ mice, the gene expression of EJ was analysed in WT mice, receiving WT, TLR4^−/−^ or CD14^−/−^ BM transplants. Mice that received TLR4^−/−^ BM displayed decreased claudin 8 and E-cadherin expression compared to mice that received WT or CD14^−/−^ BM (Fig. 6). Conversely, no differences in the occludin expression were observed.

**Figure 7:**
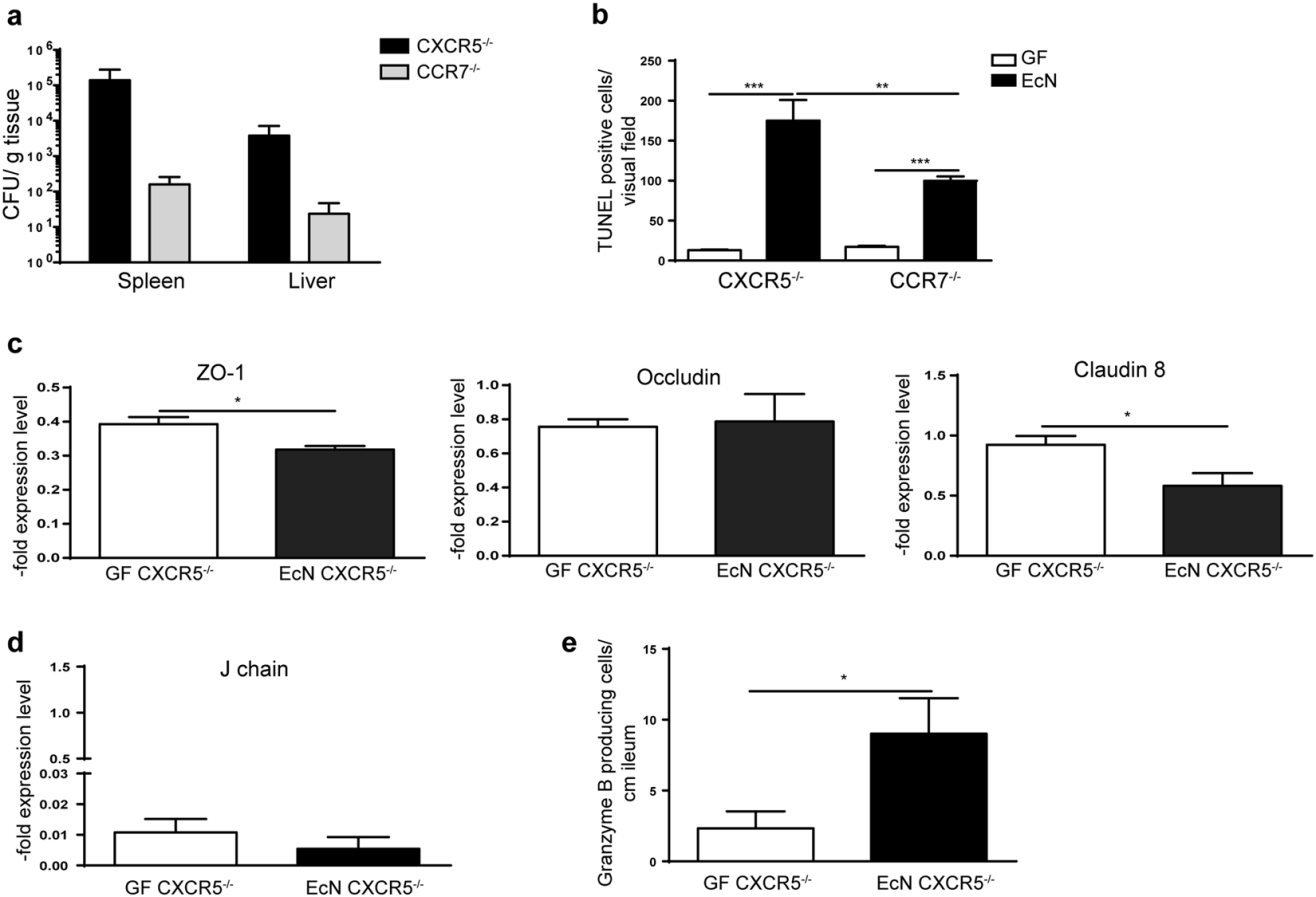
B cell contribution to the intestinal homeostasis. (**a**) Bacterial CFU determined 72 h post EcN monoassociation in spleen and liver of CXCR5^−/−^ and CCR7^−/−^ mice (n = 4). (**b**) Quantification of TUNEL stained cells in the ileum of CXCR5^−/−^ and CCR7^−/−^ mice before and after EcN stimulation (n = 4). (**c**) ZO-1, claudin 8 and occludin gene expression measured by qPCR in total RNA isolated from CXCR5^−/−^ ileum. Relative differences in gene expression were calculated by the comparative 2^−ΔΔCt^ method. Values are shown as fold induction (n = 4). (**d**) J chain gene expression measured by qPCR in the distal small intestine of CXCR5^−/−^ mice. Relative differences in gene expression were calculated by the comparative 2^−ΔΔCt^ method. Values are shown as fold induction (n = 4). (**e**) Quantification of granzyme B positive cells in the distal small intestine of CXCR5^−/−^ mice (n = 4).

In addition, EcN monoassociation of CXCR5^−/−^ mice, which are impaired in B cell gut homing, resulted in an increased bacterial invasion to spleen and liver compared to mice with impaired T cell gut homing (Fig. 7a). The level of EcN colonization was comparable among these two models (Supplementary Fig. 1d). Higher bacterial translocation correlated with increased cell death in the epithelial layer (Fig. 7b). Interestingly, mice with impaired B cell gut homing also displayed reduced gene expression of ZO-1 and claudin 8, whereas no impact on occludin gene expression was observed (Fig. 7c). Hence, the epithelial barrier tightness in mice defective in B cell homing is reduced as well. In addition, J chain expression was not upregulated, and the number of granzyme B producing cells was increased in these mice upon EcN challenge (Fig. 7d,e). Altogether, these results suggest an important role of B cells for the regulation of the EJ expression and a CD14-dependent communication pathway between B cells and epithelial cells.

## Discussion

The intestinal homeostasis is a delicate system, which depends on the strictly regulated dynamic crosstalk between IECs, LP immune cells and gut microbes. Even slight changes in this equilibrium can lead to pathological events such as development of IBD. Here, we demonstrate that the IBD candidate gene CD14 and the respective TLR4 pathway provide a link between innate and adaptive intestinal barrier mechanisms.

CD14, a co-receptor of TLR2 and 4, is involved in the recognition of antigens. Furthermore, CD14 has been shown to be protective in experimental colitis and that the predominant form of CD14 in the gut is sCD14^18,21,22^. Particularly sCD14 was discussed to be a modulator of homeostasis^27,36^. Moreover, sCD14 was found to be enriched in the breast milk and hypothesized to prevent inflammatory conditions in the neonatal intestine^26^. Furthermore, increased tissue destruction and translocation of *Shigella* was observed after CD14 inhibition^37^. In this study loss of CD14 resulted in an invasion of EcN to spleen and liver, indicating an increased intestinal permeability. As intestinal permeability is impacted by several factors such as epithelial cell integrity and the presence and distribution of epithelial junctions (EJ), these parameters were further characterized.

Tight (TJ) and apical junctions connect two adjacent epithelial cells and are important in the regulation of the paracellular permeability and prevention of luminal microbiota translocation^8^. Several studies demonstrated that EcN enhanced mucosal barrier integrity by upregulating TJ proteins such as ZO-1 and ZO-2^38–40^. In the present study, upregulated expression of ZO-1, claudin 8 and E-cadherin upon EcN colonization was observed in GF WT mice, whereas the expression of occludin was not changed. However, in TLR4^−/−^ and CD14^−/−^ mice EcN was not able to induce upregulation of EJ, indicating a leaky gut.

To elaborate whether epithelial integrity is causative for EcN dissemination in susceptible mice, WT, TLR4^−/−^ and CD14^−/−^ mice were challenged with the TLR2 agonist PCSK_4._ Activation of the TLR2 pathway has been shown to strengthen the intestinal barrier by enhancing the TJ formation^41,42^, e.g. in a *Citrobacter rodentium*-induced colitis model^43^. After TLR2 stimulation, EcN was found in the liver and spleen of TLR4^−/−^ mice. However, no bacteria were found in organs of CD14^−/−^ mice, corroborating CD14-independent transport of PCSK_4_ to the TLR2 receptor by LBP^44^. In the ileum, PCSK_4_ stimulation resulted in a strong upregulation of EJ gene expression in WT and CD14^−/−^, but not in TLR4^−/−^ mice; after combined PCSK_4_ and EcN challenge this upregulation was abrogated. Thus, differential expression of EJ is unlikely to be solely responsible for the EcN dislocation. Invasion of EcN may also be caused by increased cell death observed in TLR4^−/−^ and CD14^−/−^ mice after EcN inoculation. It was shown that TLR4 is essential for an appropriate anti-apoptotic response^45^. Moreover, a reduced number of apoptotic cells after PCSK_4_ and EcN challenge in CD14^−/−^ mice correlated with a decreased bacterial invasion; however, TLR2 stimulation cannot compensate for the TLR4 deficiency. Altogether these results indicate that the CD14-independant TLR2 stimulation provides a protective stimulus by decreasing IEC death rate and thus preventing bacterial dislocation.

In TLR4^−/−^ and CD14^−/−^ mice, upregulation of cytotoxic T cell genes and an increased presence of granzyme B positive cells in the LP accompanied increased cell death rate after EcN inoculation. It is known that granzyme B plays a key role in cytotoxic T lymphocyte (CTL) mediated targeting and elimination of pathogen invaded cells^46^. After being released from CTLs granzyme B granules are endocytosed into the target cell where they target caspase 3, which initiates the caspase cascade to DNA fragmentation and apoptosis^46,47^. This indicated that the increased cell death rate in mice lacking CD14 or TLR4 may also be induced by competent immune cells.

To determine whether the barrier maintenance was mainly controlled by hematopoietic cells, such as immune cells in the LP or by the non-hematopoietic cell compartment, such as epithelial cells, GF BM chimeras were created. Neither the hematopoietic nor the non-hematopoietic cell compartment could completely prevent bacterial translocation in this model. Hence, these data indicate a mixed contribution of hematopoietic and non-hematopoietic factors for the intestinal barrier maintenance and bacterial translocation. This finding is in line with the observation that sensitivity to EcN infection depended on the presence of enteric microbiota and on the presence of a fully competent innate and adaptive immune system^33,48^.

Protection against EcN dissemination in WT mice was associated with upregulation of genes clustered for B cell-mediated immune responses. Therefore, B cell function was further assessed in this model. Surprisingly, B cells were almost absent in the spleen and MLN of TLR4^−/−^ mice, whereas B cells were present in the organs of WT and CD14^−/−^ mice. In line with this observation, WT and CD14^−/−^ mice exhibited higher levels of IgA in the intestine than TLR4^−/−^ mice. However, IgG2a and IgM levels were also considerably reduced in CD14^−/−^ mice. More strikingly, upon EcN colonization, only WT mice showed a significant increase of IgA and IgG2a, and these differences were even more pronounced when measuring EcN specific immunoglobulins.

A pivotal parameter of B cell activation is the expression of the J chain, which is needed to form pentameric IgM and dimeric IgA. In addition, the J chain is crucial for the transport of these Ig isotypes over mucosal membranes via its interaction with the pIgR expressed on the basolateral surface of the IEC^35^. After EcN monocolonization, pIgR expression increased only in WT mice. In mice lacking CD14 or TLR4 pIgR expression was unchanged, indicating that only signalling through the complete TLR4/CD14 signalling pathway upregulates the pIgR expression and supporting the notion that the anti-inflammatory functions of secreted immunoglobulins rely on pIgR-mediated transcytosis. IgA forms a complex with the secretory component (SC), the extracellular domain of pIgR. This protects the secreted immunoglobulin A (SIgA) from degradation by host and bacterial proteases in the intestinal tract and, promotes glycan-dependent adherence of SIgA to bacteria and the neutralization of inflammatory host factors^49^. Moreover, it has been shown that members of the family Enterobacteriaceae may promote intestinal homeostasis by enhancing the expression of pIgR in IEC through their TLR4, but not TLR2 receptor ligands^50^. Furthermore, Schneeman *et al*. hypothesized that the signalling through LPS/TLR4 upregulates pIgR expression while minimizing pro-inflammatory responses^51^. This is in line with the findings of the bone marrow chimera experiments, in which non-hematopoietic cell produced CD14 was able to rescue the pIgR expression in mice with a functional TLR4. In addition, pIgR/SC knock-out mice that lack secretory IgA and IgM antibodies show a reduced epithelial barrier function and an increased uptake of antigens from food and commensal bacteria^52^. Moreover, sCD14 present in the breast milk induced B cell growth and differentiation as well as Ig secretion the neonatal gut^36^. Here, we show that these events may not only be neonate specific, but that CD14 exerts this protective role upon microbe encounter also in the adult gut.

To provide evidence regarding the protective role of B cells in this model, the CD20 antibody was utilized to deplete B cells in WT mice. After EcN challenge, bacterial dissemination into the liver was observed in contrast to untreated WT mice. The expression of J chain in the ileum of these mice was significantly reduced after CD20 treatment. In contrast, the number of granzyme B positive cells in the distal small intestine increased, demonstrating a negative correlation between B cell activity and increase of granzyme B positive cells. Though B cell depletion did not influence the epithelial cell death rate it influenced the EJ expression. In particular, EcN challenge did not upregulate the EJ expression in the absence of B cells in the gut. To our knowledge, this is the first report on B cells influencing the EJ expression. Transplantation of BM from TLR4^−/−^ mice, which display no or only marginally B cells in the gut, into WT mice corroborated this findings, as a decreased expression of two major EJ proteins, claudin 8 and E-cadherin was observed, in contrast to WT mice, which received WT or CD14^−/−^ BM. Therefore, the presence of B cells is necessary for expression of epithelial junctions, in particular for claudin 8. Additionally, WT recipients of CD14^−/−^ BM did not show dysregulated EJ expression, strengthening the hypothesis of a CD14-dependent communication pathway between B cells and epithelial cells, likely involving the soluble form.

To further support the role of B cells in this model, GF CXCR5^−/−^ and CCR7^−/−^ mice were colonized with EcN. CXCR5 is a receptor responsible for the homing of B-1 and B-2 cells, whereas CCR7 is responsible for the homing of T cells, DCs and partially B-2 cells into secondary lymphoid tissues such as Payer’s patches^53^. An increased EcN translocation to the spleen and liver and a higher rate of cell death was detected in mice with impaired B cell homing compared to those with impaired T cell homing. Furthermore, CXCR5^−/−^ mice showed low levels of J chain gene expression, reduced EJ expression and increased granzyme B expression, emphasizing the important role of B cells in the intestinal barrier maintenance.

It is intriguing to speculate that innate-like immune cells (B-1 and marginal zone B cells) contribute to the maintenance of the intestinal homeostasis in this model. These cells generate a rapid T cell-independent response to penetrating antigens^54^, and are the source of IgM in the serum and IgA in the gut^55^. These two cell sub-types provide a host’s first line of defence against diverse infections^56–58^ by generating a potent humoral response.

In summary, it was demonstrated that EcN invaded CD14^−/−^ mice analogous to TLR4^−/−^ mice and disseminated beyond the intestinal barrier. CD14^−/−^ and TLR4^−/−^ mice exhibited an impaired intestinal barrier characterized by reduced EJ gene expression, increased cell death and increased pro-inflammatory response. Moreover, CD14^−/−^ and TLR4^−/−^ mice showed low to no B cell activation and antibody secretion into the intestinal lumen upon EcN monoassociation indicating a critical role of the TLR4 pathway and its co-receptor CD14 for the function of the intestinal barrier upon colonization with gram-negative bacteria. Hence, the following protective mechanism of CD14 on the intestinal barrier is assumed. The process following EcN colonization requires epithelial cells to produce sCD14, which than alone or in complex with LPS binds to the TLR4 receptor on LP B cells and triggers signals leading to the secretion of immunoglobulins such as IgM and IgA. Secreted antibodies are transported to the gut lumen over pIgR, whose expression is dependent on the functional TLR4/CD14 pathway. Moreover, as an indirect or direct consequence of B cell activation the EJ get tighter. In this assumed meshwork, these events lead to the establishment of a homeostatic milieu and prevent inflammatory conditions in the gut. With the loss of CD14/TLR4, a cytotoxic T cell dominated immune response is initiated leading to epithelial cell death and bacterial dissemination. Finally, the protective role of CD14 as an intestinal homeostasis modulator was underlined emphasizing that as CD14 can be delivered to the tissue in a soluble form, it may represent a potential therapeutic option.

## Methods

### Mice

Germfree 8 week (wk) old C3H/HeOuJ (WT), C3H/HeJ (TLR4^−/−^), C3H/HeN.129S1-*Cd14*^*tm1Smg*^ (CD14^−/−^), B6.Cg-*Blr1*^*tm1Lipp*^/J (CXCR5^−/−^) and B6.129P2-*Ccr7*^*tm1Rfor*^/J (CCR7^−/−^) were obtained from the Central Animal Facility (Hannover Medical School, Hannover, Germany). Mice were maintained either in plastic film isolators or in static micro-isolators (gnotocages). If not stated otherwise mice were sacrificed by CO_2_ inhalation followed by exsanguination 72 h post inoculation (p.i.) and organs were collected for further analyses.

### Ethical statement

This study was conducted in accordance with German animal protection law and with the European Directive 2010/63/EU. All experiments were approved by the Local Institutional Animal Care and Research Advisory committee and permitted by the Lower Saxony State Office for Consumer Protection and Food Safety (LAVES; file number: 11/0499).

### *E. coli* Nissle 1917 colonization of germfree mice

EcN was grown overnight at 37 °C. The overnight culture was diluted 1:500, grown, harvested in the late logarithmic phase after reaching an OD_600_ = 1 (∼10^9^ CFU/ml) and centrifuged. The pellet was redissolved in 100 μl sterile PBS and administered by oral gavage under sterile conditions. Control groups received PBS only.

Colony forming units (CFU) were determined 72 h p.i. in spleen, liver, cecum and colon. To determine the number of CFU samples were weighed and homogenized in sterile PBS. Homogenates were serially diluted in PBS, and each dilution was cultured on blood agar plates and grown over 24 h at 37 °C. The number of colonies was counted and displayed as number of CFU/g tissue or content.

### TLR2 receptor stimulation

Sterilized synthetic lipopeptide Pam3Cys-SKKKK x 3HCl (PCSK_4_; EMC Microllections GmbH, Tübingen, Germany) was used as a TLR2 agonist. PCSK_4_ was prepared as described previously^41^. Briefly, mice received PCSK_4_ (150 µg/ml) for 4 days in their drinking water. 24 h after PCSK_4_ administration WT, TLR4^−/−^ and CD14^−/−^ mice were orally gavaged with 10^9^ CFU/100 µl of EcN or PBS.

### Creation of bone marrow chimeras

Donor mice were euthanized by CO_2_ inhalation followed by cervical dislocation. The bone marrow (BM) was separately isolated from tibia and femur by flushing it out with 3 ml RPMI medium. The cell suspension was passed through a cell strainer and coldly centrifuged. The pellet was resuspended in 1 ml RPMI and the cell number was determined. Recipient mice were lethally irradiated (9 Gy) in gnotocages using a linear accelerator (Synergy, Elekta, Stockholm, Sweden) and reconstituted intravenously with 100 μl of 5 × 10^6^ donor BM cells. Irradiated mice received 5 ml Cotrim K-ratiopharm®/l drinking water for 1 wk (240 mg/ 5 ml syrup, Ratiopharm GmbH, Ulm, Germany). 10 wk later mice were gavaged with 10^9^ CFU/100 μl of EcN or PBS.

### Microarray analysis

Distal small intestinal samples were taken and total RNA was isolated according to the manufacturer’s protocol (RNeasy Mini Kit, Qiagen, Hilden, Germany). The “Whole Mouse Genome Oligo Microarray 4 × 44 K v2” (G4846A, design ID 026655, Agilent Technologies, Santa Clara, USA) was utilized in this study, which covers roughly 32000 murine transcripts. For analysis two channel systems (“Dual-Colour” experimental design) was used. Total RNA was used to prepare Alexa555- (green channel) or Alexa647 (red channel)-labelled cRNA (Amino Allyl MessageAmp™ II Kit; Ambion, Invitrogen) as recommended by the manufacturer. cRNA fragmentation, hybridization and washing steps were carried-out exactly as recommended in the “Two-Color Microarray-Based Gene Expression Analysis Protocol V5.7” (Agilent Technologies). Slides were scanned on the Agilent Micro Array Scanner G2565CA (pixel resolution 5 μm, bit depth 20). Data extraction, processing and intra-array normalization of raw fluorescence intensity values were performed with the “Feature Extraction Software V10.7.3.1” by using the recommended default extraction protocol file: GE2_107_Sep09.xml. For inter-array normalization, processed fluorescence intensity values of the green channel, (gProcessedSignal or gPS) or the red channel (rPS) of all microarrays were subjected to global linear scaling, except for the first microarray of the whole series which served as the reference. All gPS and rPS values were multiplied by an array-specific scaling factor which, except for the first microarray of the whole series which served as the reference. All gPS and rPS values were multiplied by an array-specific scaling factor which was calculated by dividing the gPS 75th percentile of the first microarray (Array #1) by the gPS 75th percentile value of the particular microarray to be normalized (Array i in the formula shown below). Accordingly, inter-array normalized PS values (nPS) for all samples (microarray data sets), all probe measurements from both channels (red and green) were calculated by the following formula:

$$nP{S}_{Arrayi}=P{S}_{Arrayi}\times ({75}^{th}Percentile\,gP{S}_{Array\#1}/{75}^{th}Percentile\,gP{S}_{Arrayi})$$

Whole data were filtered for transcripts that fulfilled the following criteria: 1) arithmetic mean intensity of nPS values calculated from both channels >50 in each pair of small intestinal samples; 2) fold difference in processed signal intensities of the green and red channel >2 (upregulation) or <0.5 (downregulation) in each pair of small intestinal samples; 3) technical impairment, as defined by the entry “gIsFeatNonUnifOL OR rIsFeatNonUnifOL” (Feature extraction software) absent (=0) in each of the 4 samples analysed.

Furthermore, for the data visualization heat map was generated by use of Microsoft Excel 2010. Heat map represents fold induction – upregulation (values above 1) or downregulation (values below 1).

### Quantitative real-time PCR (qPCR)

RNA extraction from ileal samples was performed as described previously^59^. cDNA synthesis was carried out by use of the QuantiTect Reverse Transcription Kit (Qiagen, Hilden, Germany) according to the manufacturer’s protocol. QPCR was performed using QuantiTect Primer Assays for *Cd14* (Mm_Cd14_1_SG), ZO-1 (Mm_Tjp1_1_SG, Qiagen), claudin 8 (Mm_Cldn8_1_SG), occludin (Mm_Ocln_1_SG), E-cadherin (Mm_Cdh1_1_SG), J chain (Mm_Igj_1_SG) and pIgR (Mm_Pigr_1_SG). Actin (Mm_Actb_2_SG) was used as endogenous control. Detection was performed with the StepOnePlus™ Real-Time PCR System (Applied Biosystems, Weiterstadt, Germany) using the Fast SYBR Green^®^ Master Mix according to the manufacturer’s instruction. All reactions were run in triplets. The amplified PCR product was verified by melting curve analysis. Relative gene expression was calculated using the 2^−ΔΔCt^ method.

### Immunofluorescence

Immunofluorescence staining was performed on FFPE tissue sections using several primary antibodies: rabbit anti-occludin polyclonal antibody (1:100, Invitrogen, Darmstadt, Germany), rabbit anti-ZO-1 polyclonal antibody (1:50, Invitrogen), rabbit anti-granzyme B polyclonal antibody (1:100, Abcam, Cambridge, United Kingdom) and goat anti-IgR polyclonal antibody (1:250, R&D systems, Minneapolis, USA) applied by manufacturer instructions. Primary antibodies were visualized by DyLight^®^594 or DyLight^®^488 conjugated donkey anti-rabbit polyclonal secondary antibody (1:500, Abcam) and Alexa Fluor 488 conjugated donkey anti-goat polyclonal antibody (1:500, Abcam). Nuclear counterstaining was performed with a mounting medium containing DAPI (Vectashield, Vector Laboratories, USA) and examined using the Zeiss Axioskop 40 microscope (Carl Zeiss Microscopy GmbH, Göttingen, Germany) connected to an AxioCam MRm (Carl Zeiss). Number of granzyme B cells was determined by counting positive cells per 1 cm ileal tissue.

### Terminal deoxynucleotidyl transferase dUTP Nick-End Labeling (TUNEL) assay

Intestinal cells undergoing cell death were quantified by TUNEL staining (*In situ* Cell Death Detection Kit, TMR red, Roche, Germany) according to the supplier’s protocol. DAPI staining was applied to visualize nuclei. Samples were prepared in duplicates. Cell death rate was determined by two independent examiners counting positive cells per visual field (ten fields per slide). Tissue sections were examined as described in the previous section.

### Enzyme-linked immunosorbent assay (ELISA)

Secreted antibodies in the intestinal lavage were analysed using the Mouse Immunoglobulin Isotyping ELISA Kit (BD Biosciences, Heidelberg, Germany) applied as recommended by manufacturer. For the EcN specific ELISA a 96-well plate was coated with heat inactivated EcN (1:10). EcN was fixed with 0.025% glutaraldehyde for 10 min at room temperature (RT) followed by short washing with distilled water. The plate was blocked with 5% BSA in PBST (used as a sample diluent as well) for 1 h at RT. Between each step the plate was washed 4-5 times with PBST. Samples (1:20) were mounted on the plate for 2 h at 37 °C. For detection AB Biotin Rat Anti Mouse IgA (1:200), IgM (1:200) and IgG2a (1:125) were used (BD Pharmingen). Antibodies were incubated for 1 h at 37 °C. Subsequently Streptavidin HRP Enzyme (1:1000; BD Pharmingen) was added and incubated for 1 h at RT in the dark. As a substrate for the enzyme TMB Substrate Reagent Set (1:2; BD Pharmingen) was used for 10–20 min at RT in the dark. The reaction was stopped using sulfuric acid (1 M). The absorbance was read at 450 nm in the VICTOR^TM^ X3 Multilabel Plate Reader (PerkinElmer, Waltham, MA, USA).

### Western blot

For the western blot analysis total protein extracted from ileal tissue was used. Proteins were separated by sodium dodecyl sulphate polyacrylamide gel electrophoresis and transferred to a nitrocellulose membrane (Millipore). For protein detection an anti-claudin 8 primary antibody (Invitrogen, Carlsbad, CA, USA) was used. GAPDH (GenScript USA Inc., NJ, USA) was used as an internal control. All antibodies were used as recommended by manufacturer. The blots were visualized using ChemiDoc™ Touch Imaging System (Bio-Rad, Hercules, CA, USA). The protein expression was evaluated by Image Lab™ Software (Bio-Rad).

### B cell depletion

B cells were depleted using CD20 antibody (2.44 mg/ml; 1:10) generously provided by Genetech (San Francisco, USA). Each mouse received 250μg/ 100μl CD20 antibody i.p. After 7 days MLN and spleen B cells were isolated and analysed by flow cytometry.

### Flow cytometry

Cells analysed were isolated from MLN, spleen or ileum. For staining cell numbers were adjusted to 1 × 10^6^ cells/well. Cells were stained with IgM-APC-Cy7 (1:1000; Biolegend, San Diego, CA, USA), CD19-AF700 (1:1000; Biolegend), B220-VioBlue (1:500, Miltenyi Biotec GmbH, Bergisch Gladbach, Germany), granzyme B-FITC (1:100; eBioscience, San Diego, CA, USA), CD8-PE-Cy7 (1:3000; Biolegend) or CD3-APC-Cy7 (1:1000; Biolegend) antibody. For all dilutions MACS buffer was used. Cells were analysed in the Gallious Flow Cytometer (Beckman Coulter).

### Statistical analysis

If not stated otherwise values are means ± standard error of the mean (SEM). All statistical analyses were performed by use of GraphPad Prism6® software (GraphPad Software, La Jolla, USA). For parametric data, t-tests and one way analysis of variance (ANOVA) with Tukey’s test as post-hoc test were carried out. *P* < 0.05 was considered significant (*P < 0.05; **P < 0.01; ***P < 0.001; ****P < 0.0001).

### Data availability

The datasets generated during and/or analysed during the current study are available from the corresponding author on reasonable request.

## Electronic supplementary material

Supplementary information
