# Supplementary material for: Loss of CD14 leads to disturbed epithelial-B cell crosstalk and impairment of the intestinal barrier after E. coli Nissle monoassociation
Source: Sci Rep. 2018 Jan 15;8:719. doi: 10.1038/s41598-017-19062-7 (PMC5768714; doi:10.1038/s41598-017-19062-7)
Supplement: Supplementary file 1 — Supplementary information [file 41598_2017_19062_MOESM1_ESM.pdf]

Loss of CD14 leads to disturbed epithelial-B cell crosstalk and impairment of the intestinal barrier after *E. coli* Nissle monoassociation

Marijana Basic, Manuela Buettner, Lydia M. Keubler, Anna Smoczek, Inga Bruesch, Stephanie Buchheister, and André Bleich

Supplementary Figure S1

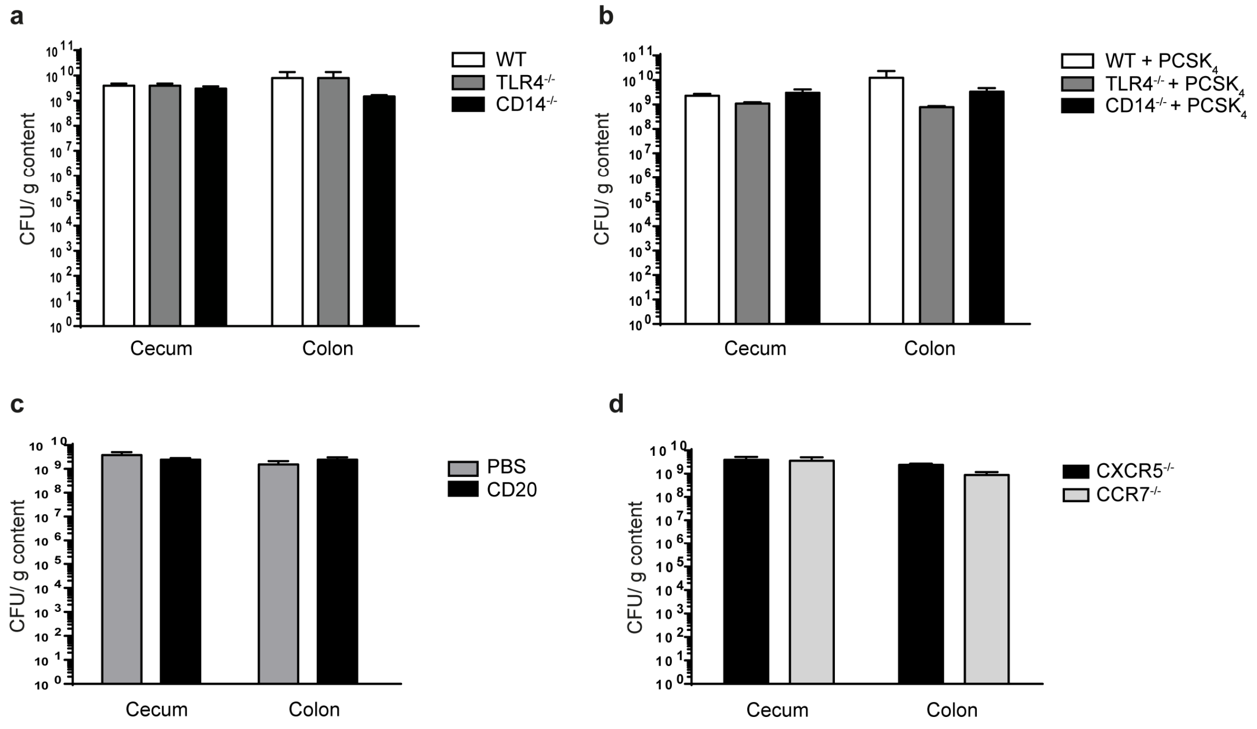

EcN gut colonization rate

a. Bacterial CFU determined 72h post EcN monoassociation in cecum and colon of WT, $TLR4^{-/-}$ and $CD14^{-/-}$ mice (n=4-5).

b. Bacterial CFU determined 72h post EcN monoassociation and $PCSK_4$ challenge in cecum and colon of WT, $TLR4^{-/-}$ and $CD14^{-/-}$ mice (n=5-6).

c. Bacterial CFU determined 72h post EcN monoassociation in cecum and colon of WT, $TLR4^{-/-}$ and $CD14^{-/-}$ mice with or without CD20 treatment (n=5).

d. Bacterial CFU determined 72h post EcN monoassociation in cecum and colon of $CXCR5^{-/-}$ and $CCR7^{-/-}$ mice (n=4).

Supplementary Figure S2

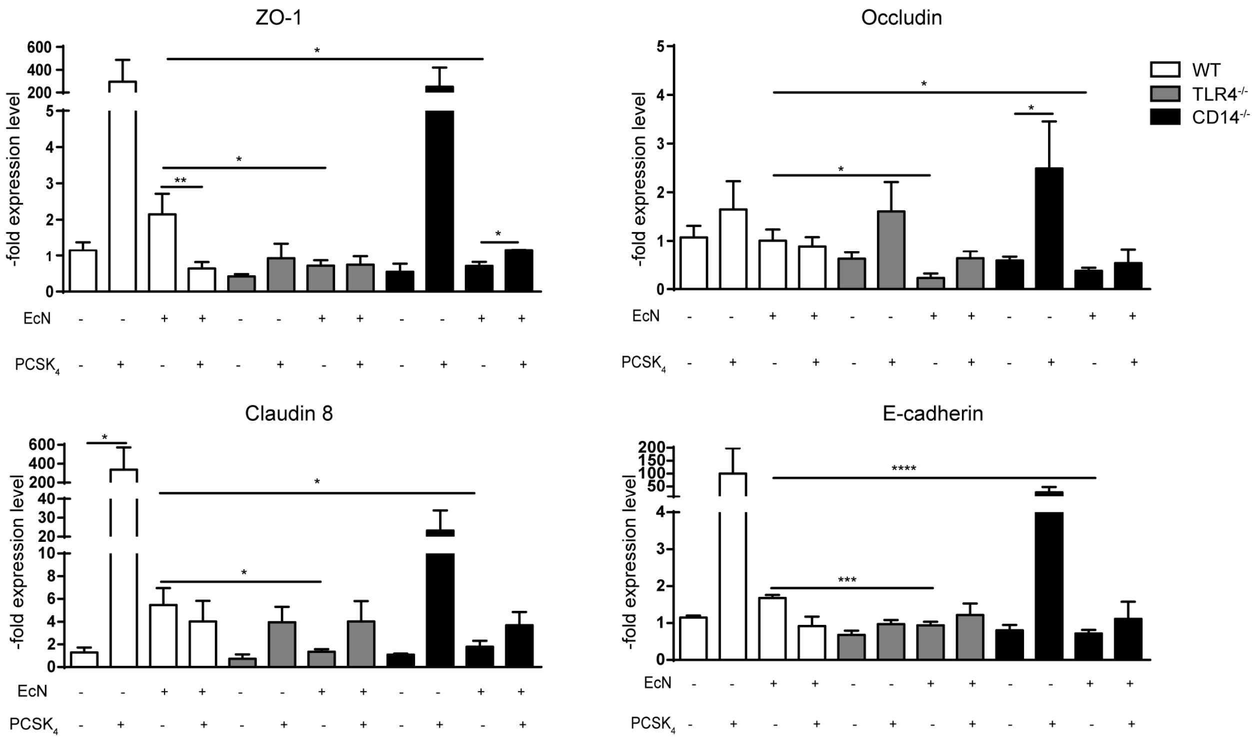

Influence of PCSK$_4$ treatment on the expression of epithelial junctions after EcN challenge

Gene expression of ZO-1, occludin, claudin 8 and E-cadherin measured by qPCR in total RNA isolated from ileum. Relative differences in gene expression were calculated by the comparative $2^{-\Delta\Delta Ct}$ method. Values are shown as fold induction (n=4-6).

Supplementary Figure S3

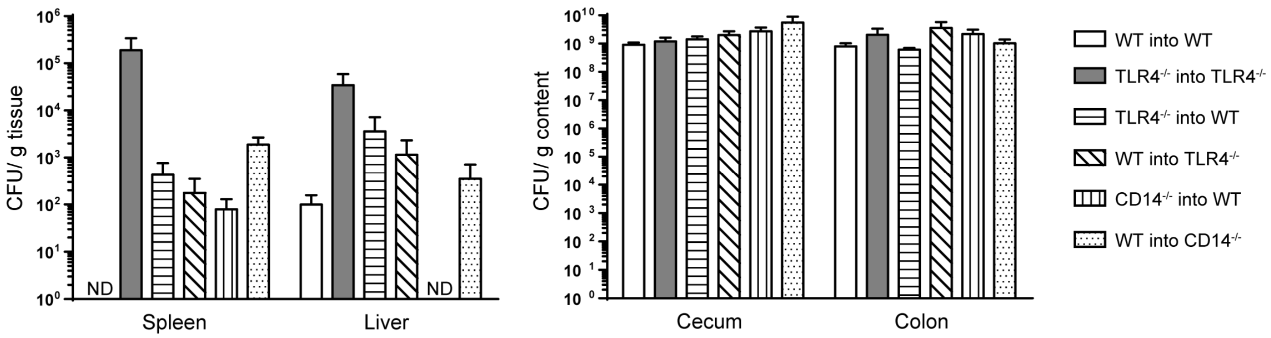

Bacterial translocation in BM chimeras

Bacterial CFU determined 72h post EcN monoassociation in spleen, liver, cecum and colon content (n=4-5). ND-not detected
